# The Outcome of Induction Therapy for EBV-Related Hemophagocytic Lymphohistiocytosis: A Model for Risk Stratification

**DOI:** 10.3389/fimmu.2022.876415

**Published:** 2022-07-04

**Authors:** Tingting Cui, Jingshi Wang, Zhao Wang

**Affiliations:** Department of Hematology, Beijing Friendship Hospital, Capital Medical University, Beijing, China

**Keywords:** hemophagocytic lymphohistiocytosis, Epstein–Barr virus, outcome, induction therapy, predicting model

## Abstract

**Background:**

Epstein–Barr virus (EBV)–related hemophagocytic lymphohistiocytosis (HLH) is an abnormal inflammation caused by EBV infection, which has high mortality during induction therapy.

**Objectives:**

This study is aimed to build a model to predict the risk of death during induction therapy.

**Methods:**

The patients with EBV-HLH admitted from January 2015 to December 2018 were retrospectively reviewed. The primary outcome was death during induction therapy. The interval from receiving therapy to death or the end of induction therapy was the observing time. The patients admitted from January 2015 to December 2017 were assigned to the primary group, and the patients admitted from January to December 2018 were assigned to the validation group.

**Results:**

We included 234 patients with EBV-HLH, of whom 65 (27.4%) died during induction therapy. The middle observing time was 25 days. On the basis of the primary group, the multivariate Cox analysis demonstrated age >18 years, blood urea nitrogen, procalcitonin >2 µg/L, serum CD25, and EBV-DNA in peripheral blood mononuclear cell as the risk factors of death during induction therapy. We developed a nomogram integrating the above factors with high predictive accuracy (c-statistic, 0.86) and stratified all patients into the high-risk and the low-risk groups. On the basis of the validation group, the high-risk patients had a higher risk of death (hazard ratio, 4.93; *P =* 0.012). In the subgroup analysis based on patients receiving etoposide-based strategy, the mortality in high-risk and low-risk patients was 43.9 and 3.1 per 100 person-weeks, respectively.

**Conclusion:**

We developed a nomogram for risk stratification of patients with EBV-HLH receiving induction therapy.

## Introduction

Epstein–Barr virus (EBV)–related hemophagocytic lymphohistiocytosis (HLH) has been found as an abnormal inflammation, characterized by the overactivity of immune cells caused by EBV infection. Without appropriate therapy, patients with EBV-HLH have a mortality up to 20%–95.7% (1–3). Although the allogenic haemopoietic stem cell transplantation has been found as an effective method to treat EBV-HLH, about two-thirds of patients will die during induction therapy, especially in the sixth to the eighth week after they receive standard therapy (2). To identify the patients with a high risk of death during induction therapy is conducive to managing the patients with EBV-HLH and predicting the outcome.

There have been few reliable models and factors to predict the risk of death of patients with EBV-HLH after they receive induction therapy. Moreover, the factors of the death of patients with EBV-HLH during induction therapy were also unclear. Some studies found that age, serum CD25 (sCD25), and EBV loading were the factors of poor outcome (4–6). However, the correlation between these factors and death during induction therapy has been unknown.

In this study, we reviewed the clinical characteristics and therapy strategy of patients with EBV-HLH admitted to our medical institution. This study is aimed to investigate the risk factors of the death during induction therapy and build a model to predict this event.

## Methods and Materials

### Population and Study Design

The study was approved by the institutional review board of Beijing Friendship Hospital (2020-P2-096-01). Written informed consents were obtained, and the privacy of patients was effectively protected.

In this study, the patients with EBV-HLH admitted to our medical institution between January 2015 and December 2018 were retrospectively reviewed. The inclusion criteria of patients with EBV-HLH included the following (1): patients were diagnosed with HLH in accordance with HLH-2004 diagnostic criteria; (2) the EBV-DNA was positive (>500 copies/ml) in plasma or peripheral blood mononuclear cell (PBMC); and (3) complete or traceable clinical record. A total of 270 eligible patients were included. Moreover, 30 patients diagnosed with lymphoma by pathological examinations and (2) six patients diagnosed with primary HLH by gene examination were excluded [We excluded four patients with familial hemophagocytic lymphocytosis 2 (compound heterozygote). One patient with familial hemophagocytic lymphocytosis 3 and one patient with X-linked lymphoproliferative disease were excluded. No primary immunodeficiency]. Last, we included 234 appropriate patients with EBV-HLH.

**Figure 1:**
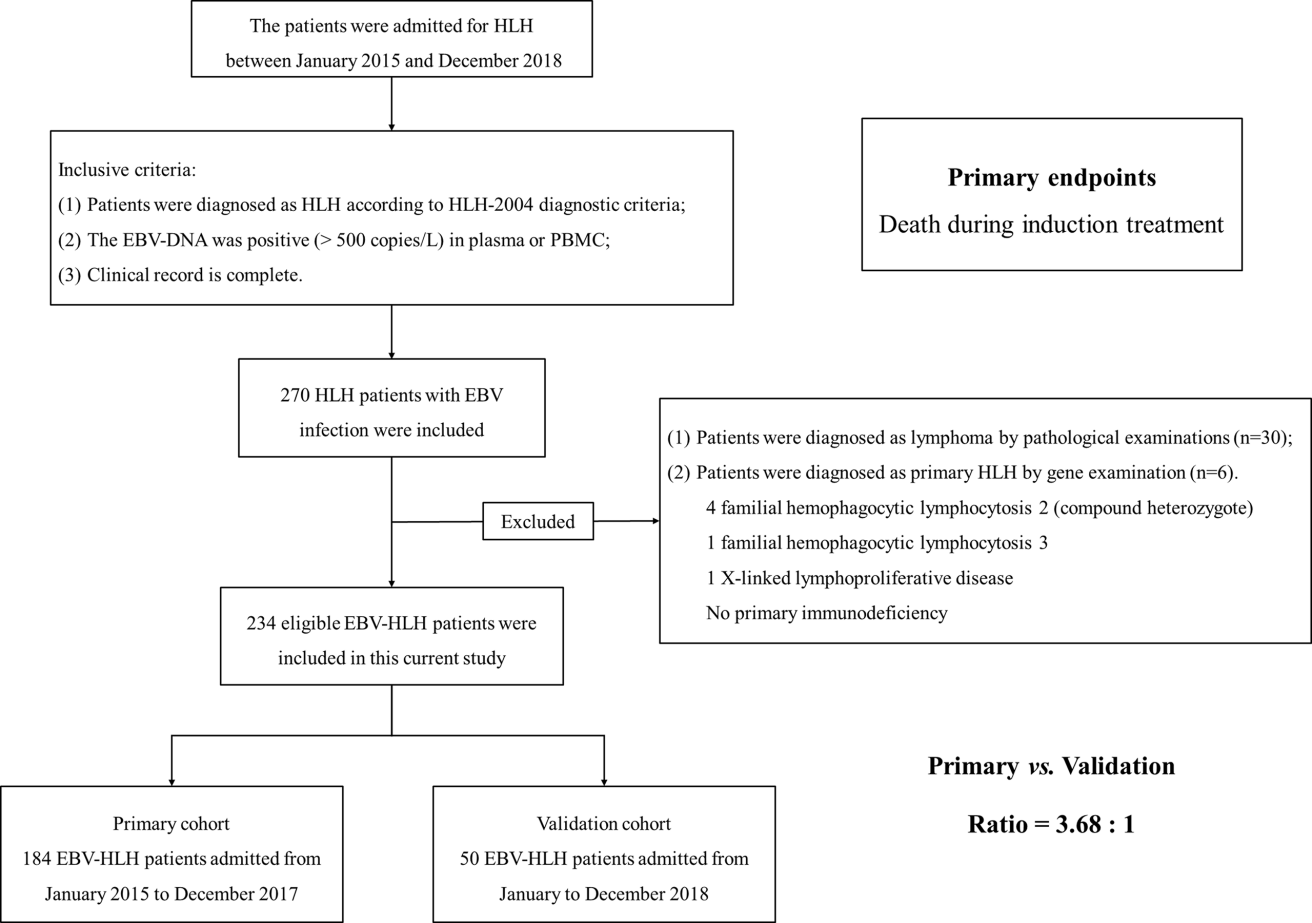
The flow chart of patient enrollment. We retrospectively reviewed the patients admitted to our medical institution from January 2015 to December 2018. Finally, 234 patients were enrolled into this current study. We set patients admitted from January 2015 to December 2017 as primary cohort and patients admitted from January to December 2018 as validation cohort. The ratio of primary cohort to validation cohort was approximately 3.5:1.

Among the included patients with EBV-HLH, the patients admitted from January 2015 to December 2017 were assigned to the primary group (184 patients with EBV-HLH) to build a predictive model, and the patients admitted from January to December 2018 were assigned to the validation group (50 patients with EBV-HLH). The ratio of the primary group to the validation group was about 3.5:1 (**Figure 1**).

### Procedures

We acquired the information according to the electronic medical records, including demographic information (e.g., gender, age, the history of infection, the history of tuberculous infection and bleeding history, brain involvement, and liver/spleen involvement), laboratory findings at admission [i.e., white blood cell (WBC) count, hemoglobin, platelet count, glutamic-pyruvic transaminase (ALT), glutamic oxalacetic transaminase (AST), gamma-glutamyl transpeptidase (GGT), alkaline phosphatase (ALP), total bilirubin (Tbi), direct bilirubin (Dbi), indirect bilirubin (Ibi), albumin (ALB), creatinine (Cr), blood urea nitrogen (Bun), K^+^, Ca^2+^, Na^+^, fibrinogen, procalcitonin (PCT), and erythrocyte sedimentation rate (ESR)], and specific findings [hemophagocytosis, serum ferritin, sCD25, the activity of nature killer cells (NK cells), EBV-DNA in plasma and PBMC, the CD107a in NK cell (NK-CD107a), and the CD107a in cytotoxic T lymphocyte (CTL-CD107a)].

We divided all the patients into the group with the age >18 years and the group ≤18 years. For the history of tuberculous infection correlated with poor outcome in HLH, patients were categorized as the group of history of tuberculous infection and the group of other infections. According to liver and spleen involvement, patients were classified as splenomegaly, hepatomegaly, and hepatosplenomegaly groups. PCT >2 µg/L indicated a severe inflammation, so we classified patients into PCT >2 µg/L and PCT ≤2 µg/L groups.

### Management and Induction Therapy

In accordance with HLH therapy guideline, the therapy strategy for patients with EBV-HLH included etoposide-based protocol (e.g., HLH-1994, HLH-2004, doxorubicin-etoposide-methylprednisolone (DEP), and PEG aspargase-DEP (LDEP)) instead of etoposide-based protocol (e.g., corticosteroids and corticosteroids + rituximab) (7).

In general, the interval from standard induction therapy to transplantation is at least 8 weeks. Moreover, death occurred mostly in the sixth to the eighth week (2). For the above reasons, we observed patients and recorded the outcome at weeks 4 and 8 after standard therapy began.

### Outcomes

The primary outcome was death during induction therapy (in 8 weeks after the patients received standard therapy). We set the interval from receiving standard therapy to death or the end of induction therapy (8 weeks) as the observing time for further analysis. According to the primary outcome, we divided all patients into the survival group and the death group.

### Statistical Analysis

Continuous variables with normal distribution were expressed as means and standard deviation, or medians (m) and inter-quartile range (IQR) if otherwise. Categorical variables were expressed as numbers (no.) and percentage (%). We compared the differences between the death and survival groups in continuous variables through student’s t-tests or Wilcoxon rank sum tests, and we compared the differences in categorical variables through chi-square tests or Fisher’s exact tests. We conducted univariate Cox regression analyses to detect the factors for the primary outcome. We entered the parameters with statistical significance in univariate Cox regression analyses in multivariate regression models through a backward step-down selection process. The result was expressed as hazard ratios (HR) and 95% confidence intervals (CI).

The nomogram was developed with the package of “rms” in R version 4.1.1. Subsequently, we generated a nomogram to predict the primary outcome (death during induction therapy). In accordance with the result of the multivariate Cox regression analysis, we developed a nomogram model that included the factors for the primary outcome. We calculated the survival probabilities 4 and 8 weeks after the patients received standard therapy. We measured the performance of the nomogram to predict the primary outcome by the c-statistic values in receiver operating the characteristic curve (ROC) analysis. The model with c-statistic >0.8 and *P <*0.05 was considered to be good clinical utility. The calibration curves were generated to evaluate the calibration of the nomogram. We performed bootstrapping validation for the nomogram (1,000 bootstrap resamples). To identify the breakpoint of nomogram points for the primary outcome, we conducted regression discontinuity analysis using the package of “smoothHR” and “Hmisc” in R version 4.1.1. The restricted cubic splines were used to smooth model and visualize the relation of predicted death risk and actual death risk. The nomogram point value with breaking HR change was recognized as the breakpoint. According to the calculated breakpoint, we separated all patients into the low-risk group and the high-risk group. We conducted the survival analyses using Kaplan–Meier method. Moreover, the mortality was calculated.

The statistical analysis was conducted using SPSS 24.0 (SPSS, Chicago, IL), and two-sided *P <*0.05 had statistical significance.

## Results

### Demographic and Clinical Characteristics of Patients in the Primary Group

Among 184 patients with EBV-HLH included in the primary group, 54 died (29.3%) during induction therapy. Twenty-two patients died of multiple organ dysfunction syndrome, 23 patients died of infection, six patients died of gastrointestinal bleeding, and three patients died of central nervous system involved. Among 50 patients with EBV-HLH included in the validation group, 10 died (20%) during induction therapy, three patients died of multiple organ dysfunction syndrome, five patients died of infection, one patient died of gastrointestinal bleeding, and one patient died of central nervous system involved. **Table 1** lists the demographic and clinical information of patients with EBV-HLH in the primary group. One-hundred fifteen patients were male, with a median age of 25.0 (15.5–39.5). One-hundred forty-seven (79.9%) patients were treated with etoposide. More patients with age >18 years (*P =* 0.007), history of tuberculous infection (*P =* 0.043), brain involvement (*P* < 0.001), hepatosplenomegaly (*P =* 0.049), and PCT >2 µg/L (*P =* 0.027) died during induction therapy. Moreover, sCD25 level and EBV-DNA in PBMC were higher in the patients ending up dead (*P =* 0.019 and 0.007, respectively). Moreover, the significance was found in WBC count (*P =* 0.015), hemoglobin (*P =* 0.001), platelet count (*P =* 0.007), GGT (*P =* 0.007), ALP (*P =* 0.049), Tbi (*P =* 0.031), Dbi (*P =* 0.004), ALB (*P* < 0.001), Bun (*P =* 0.038), Ca^2+^ (*P =* 0.002), Na^+^ (*P =* 0.013), and fibrinogen (*P =* 0.018) between the death group and the survival group. However, there was no significant difference in gender, therapy strategy, other infections, splenomegaly, hepatomegaly, ALT, AST, Ibi, Cr, K^+^, ESR, hemophagocytosis, serum ferritin, the activity of NK cells, EBV-DNA in plasma, and CD107a (all *P >*0.05).

**Table 1:** Demographic and clinical information of patients with EBV-HLH in the primary cohort.

Characteristics	Deathn = 54	Survivaln = 130	*P*-value
Male, no. (%)	29 (53.7%)	86 (66.2%)	0.113
Age >18 years, no. (%)	44 (81.4%)	79 (60.8%)	0.007^┼^
Therapy strategy, no. (%)			0.206
Etoposide-based	40 (74.1%)	107 (82.3%)	
No etoposide-based	14 (25.9%)	23 (17.7%)	
Infection history, no. (%)			
Tuberculous	12 (22.2)	14 (10.8%)	0.043^┼^
Other	50 (92.5%)	111 (85.4%)	0.179
Brain involvement, no. (%)	18 (33.3%)	12 (1.5%)	<0.001^┼^
Liver/spleen involvement, no. (%)			
Splenomegaly	18 (33.3%)	60 (46.2%)	0.110
Hepatomegaly	1 (1.9%)	4 (3.1%)	0.643
Hepatosplenomegaly	28 (51.9%)	47 (36.2%)	0.049^┼^
Laboratory findings			
WBC count, m (IQR), ×10^9^	1.5 (0.9–2.5)	2.1 (1.4–3.6)	0.015^┼^
Hemoglobin (g/l), m (IQR)	87 (77–100)	101.5 (84–119)	0.001^┼^
Platelet count, m (IQR), ×10^9^	45 (24–68)	60.5 (37–118)	0.007^┼^
ALT (U/L), m (IQR)	91.5 (63.0–183.0)	81.5 (42.0–188.0)	0.455
AST (U/L), m (IQR)	145.2 (52.5–257.0)	84.9 (43.0–193.0)	0.070
GGT (U/L), m (IQR)	202 (111–378)	124 (48–282)	0.007^┼^
ALP (U/L), m (IQR)	290 (129–566)	207 (100–400)	0.049^┼^
Tbi (μmol/L), m (IQR)	32.0 (14.2–62.4)	19.4 (12.3–34.0)	0.031^┼^
Dbi (μmol/L), m (IQR)	17.2 (5.0–58.1)	7.4 (3.5–20.0)	0.004^┼^
Ibi (μmol/L), m (IQR)	12.9 (8.3–27.3)	12.0 (7.1–15.5)	0.055
ALB (g/L), m (IQR)	27.2 (24.0–32.9)	31.7 (27.7–37.4)	<0.001^┼^
Cr (μmol/L), m (IQR)	55.2 (44.0–78.3)	53.7 (44.2–69.4)	0.455
Bun (mmol/L), m (IQR)	5.7 (4.2–7.5)	5.0 (3.7–7.1)	0.038^┼^
K^+^ (mmol/L), m (IQR)	3.92 (3.61–4.10)	4.04 (3.72–4.39)	0.052
Ca^2+^ (mmol/L), m (IQR)	1.97 (1.78–2.11)	2.10 (1.93–2.23)	0.002^┼^
Na^+^ (mmol/L), m (IQR)	134.0 (132.1–137.8)	136.9 (133.2–139.6)	0.013^┼^
Fibrinogen (g/L), m (IQR)	1.26 (0.77–1.79)	1.61 (1.02–2.47)	0.018^┼^
PCT > 2 µg/L, no. (%)	15	16	0.027^┼^
ESR (mm/H), m (IQR)	27 (10–30)	18 (10–30)	0.845
Specific findings			
Hemophagocytosis, no. (%)	41 (75.9%)	105 (80.8%)	0.461
Serum ferritin (ng/ml), m (IQR), ×10^3^	2.82 (1.65–15.00)	2.20 (0.95–13.07)	0.078
sCD25 (pg/ml)^†^, m (IQR), ×10^4^	2.52 (1.94–4.40)	2.18 (0.31–4.01)	0.019^┼^
The activity of NK cells (%)^‡^, m (IQR)	14.4 (13.0–14.5)	14.4 (13.5–16.4)	0.085
EBV-DNA (copies/ml), m (IQR), ×10^5^			
Plasma	3.8 (1.5–17.0)	3.8 (0.3–26.0)	0.530
PBMC	8.7 (4.6–100.0)	6.2 (0.8–21.0)	0.007^┼^
NK-CD107a (%), m (IQR)	12.2 (10.4–12.2)	12.2 (10.7–20.3)	0.198
CTL-CD107a (%), m (IQR)	2.7 (2.7–3.4)	2.7 (2.2–3.3)	0.247

^┼^The parameter was significant.

^†^The normal range of sCD25 is <6,400.

^‡^The normal range of the activity of NK cells is ≥15.11%.

WBC, white blood cell; ALT, glutamic-pyruvic transaminase; AST, glutamic oxalacetic transaminase; GGT, gamma-glutamyl transpeptidase; ALP, alkaline phosphatase; Tbi, total bilirubin; Dbi, direct bilirubin; Ibi, indirect bilirubin; ALB, albumin; Cr, creatinine; Bun, blood urea nitrogen; PCT, procalcitonin; ESR, erythrocyte sedimentation rate; serum CD25; NK cells, nature killer cells; EBV, Epstein–Barr virus; DNA, deoxyribonucleic acid; PBMC, peripheral blood mononuclear cell; CTL, cytotoxic T lymphocyte.

### The Factors of Death During Induction Therapy and Nomogram

**Figure 2:**
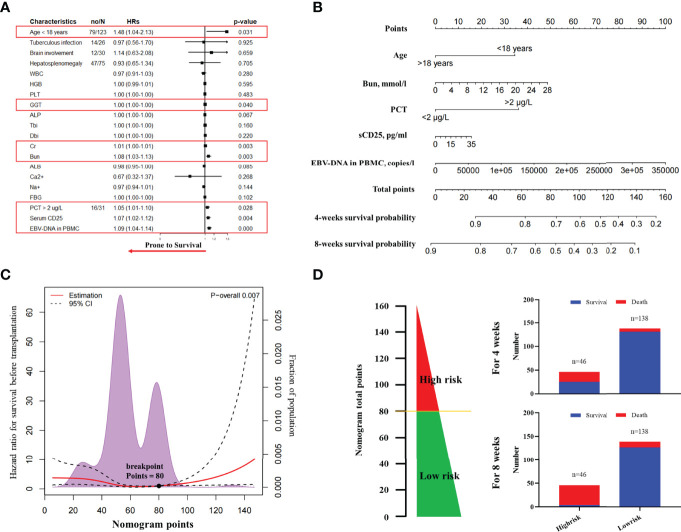
The nomogram to predict the risk of death in induction therapy. **(A)** The forest plot of univariate Cox regression analysis. The result showed that age, GGT, Cr, Bun, PCT, sCD25, and EBV-DNA in PBMC were risk factors associated with the death in induction therapy. **(B)** The nomogram to predict the risk of death in induction therapy. **(C)** The breakpoints of nomogram points for the death in induction therapy. Using the regression discontinuity analysis, we identified the breakpoints of nomogram points as 80. **(D)** We categorized 46 patients as the high-risk group and 138 patients as the low-risk group. The histogram showed the death rate in each group. HR, hazard ratio; WBC, white blood cell; GGT, gamma-glutamyl transpeptidase; ALP, alkaline phosphatase; Tbi, total bilirubin; Dbi, direct bilirubin; ALB, albumin; Cr, creatinine; Bun, blood urea nitrogen; PCT, procalcitonin; EBV, Epstein–Barr virus; PBMC, peripheral blood mononuclear cell; CTL, cytotoxic T lymphocyte.

On the basis of the primary group, we entered all significant parameters into the univariate analysis and entered the parameters with clinical significance into the univariate Cox regression analysis. As indicated by the result, age >18 years (HR, 1.48; 95% CI, 1.04–2.13; *P =* 0.031), GGT (HR, 1.00; 95% CI, 1.00–1.00; *P =* 0.040), Cr (HR, 1.01; 95% CI, 1.00–1.01; *P =* 0.003), Bun (HR, 1.08; 95% CI, 1.03–1.13; *P =* 0.003), PCT >2 µg/L (HR, 1.05; 95% CI, 1.01–1.10; *P =* 0.028), sCD25 (HR, 1.07; 95% CI, 1.02–1.12; *P =* 0.004), and EBV-DNA in PBMC (HR, 1.09; 95% CI, 1.04–1.14; *P* < 0.001) were correlated with death during induction therapy (**Figure 2A** and **Supplementary Table 1**). Subsequently, we entered the above parameters into the multivariate Cox regression analysis. The parameters, including age >18 years (HR, 1.24; 95% CI, 1.08–1.42; *P =* 0.040), Bun (HR, 1.08; 95% CI, 1.02–1.13; *P =* 0.015), PCT > 2 µg/L (HR, 2.60; 95% CI, 1.49–4.55; *P =* 0.001), sCD25 (HR, 1.08; 95% CI, 1.02–1.14; *P =* 0.010), and EBV-DNA in PBMC (HR, 1.10; 95% CI, 1.05–1.16; *P* < 0.001), were independent risk factors of death during induction therapy (**Table 2**).

**Table 2:** Multivariate Cox regression analysis^†^ for the risk factors associated with the survival in induction therapy based on primary cohort.

	HR	95% CI	*P*-value
Age >18 years	1.24	1.08–1.42	0.040
GGT	Omitted		
Cr	Omitted		
Bun	1.08	1.02–1.13	0.015
PCT >2 µg/L	2.60	1.49–4.55	0.001
sCD25, ×10^4^	1.08	1.02–1.14	0.010
EBV-DNA in PBMC, ×10^5^	1.10	1.05–1.16	<0.001

^†^The multivariate Cox regression analysis was performed using backward method.

HR, hazard ratio; GGT, gamma-glutamyl transpeptidase; Cr, creatinine; Bun, blood urea nitrogen; PCT, procalcitonin; serum CD25; EBV, Epstein–Barr virus; DNA, deoxyribonucleic acid; PBMC, peripheral blood mononuclear cell.

**Figure 3:**
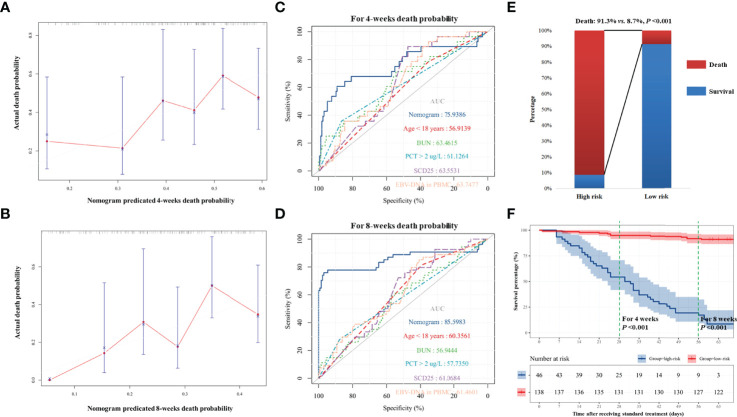
The predicting accuracy of nomogram for death in induction therapy. **(A, B)** For death within 4 and 8 weeks, the nomogram has good consistency with actual death. **(C, D)** The predictive accuracy of nomogram and other risk factors for 4-week death and 8-week death. **(E)** The proportion of death in the high-risk group and the low-risk group. The difference was significant (91.3% vs. 8.7%, *P* < 0.001). **(F)** The survival analysis showed that high-risk patients had higher risk of death. Bun, blood urea nitrogen; PCT, procalcitonin; EBV, Epstein–Barr virus; DNA, deoxyribonucleic acid; PBMC, peripheral blood mononuclear cell.

We developed a nomogram integrating the above risk factors (**Figure 2B**), which can predict the survival probability of patients with EBV-HLH during induction therapy. In accordance with the result of the regression discontinuity analysis (**Figure 2C**), we identified the breakpoints of the nomogram points as 80. According to **Figure 2D**, we divided all patients into the low-risk group (>80 points) and the high-risk group (≤80 points). The calibration curves indicated that the death predicted by the nomogram was consistent with the actual death (**Figures 3A, B**). The prediction accuracy of the nomogram was higher than that of any single parameters for the death during induction therapy (AUC was 0.76 for the 4-week death and was 0.86 for the 8-week death; **Figures 3C, D** and **Table 3**). The proportion of death was significantly different between the high-risk group and the low-risk group (**Figure 3E**). The survival analysis indicated that the high-risk group had a high risk of death during induction therapy (**Figure 3F**).

**Table 3:** The predicting accuracies of different models for the death after receiving standard therapy.

Models	Survival within 4 weeks				Survival within 8 weeks			
	Primary cohort		Validation cohort		Primary cohort		Validation cohort	
	c-statistic values	*P*-value	c-statistic values	*P*-value	c-statistic values	*P*-value	c-statistic values	*P*-value
Age >18 years	0.57 (0.46–0.68)	0.245	0.62 (0.45–0.81)	0.235	0.60 (0.52–0.69)	0.027	0.63 (0.40–0.84)	0.399
Bun	0.63 (0.52–0.75)	0.023	0.80 (0.59–0.97)	0.016	0.57 (0.48–0.66)	0.138	0.76 (0.61–0.90)	0.032
PCT >2 µg/L	0.61 (0.49–0.73)	0.061	0.82 (0.62–0.95)	0.219	0.58 (0.48–0.67)	0.099	0.63 (0.52–0.74)	0.414
sCD25	0.64 (0.54–0.73)	0.024	0.83 (0.74–0.90)	0.013	0.61 (0.53–0.69)	0.019	0.77 (0.68–0.98)	0.017
EBV-DNA in PBMC	0.64 (0.54–0.74)	0.021	0.56 (0.36–0.72)	0.874	0.61 (0.53–0.70)	0.014	0.51 (0.35–0.72)	0.649
Nomogram	0.76 (0.72–0.87)	<0.001	0.85 (0.72–0.93)	<0.001	0.86 (0.74–0.95)	0.004	0.81 (0.71–0.94)	0.011

Bun, blood urea nitrogen; PCT, procalcitonin; serum CD25; EBV, Epstein–Barr virus; DNA, deoxyribonucleic acid; PBMC, peripheral blood mononuclear cell.

### Validation of the Nomogram and Subgroup Analysis

The prediction accuracy of the nomogram was subsequently validated in validation group. The comparison of characteristics between the primary group and validation group is summarized in **Supplementary Table 2**.

**Figure 4:**
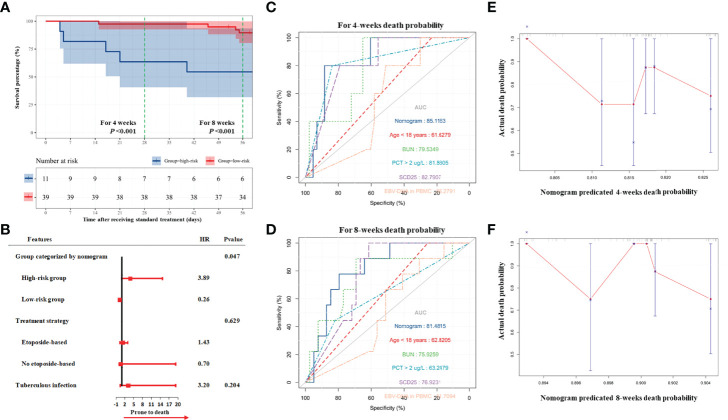
Validation of nomogram for death in induction therapy. **(A)** On the basis of validation cohort, the survival analysis showed that high-risk patients had higher risk of death. **(B)** The result of multivariate Cox analysis. The result showed that the high-risk group categorized by nomogram was related to high risk of death. **(C, D)** The predicted accuracy of nomogram and other risk factors for 4- and 8-week death. **(E, F)** The calibration analyses showed that the death predicted by nomogram was consistent with the actual death. Bun, blood urea nitrogen; PCT, procalcitonin; EBV, Epstein–Barr virus; DNA, deoxyribonucleic acid; PBMC, peripheral blood mononuclear cell.

According to the result of the validation group, the high-risk group still had a higher risk of death (HR, 4.93; 95% CI, 1.42–17.11; *P =* 0.012) during induction therapy (**Figure 4A**). As demonstrated by the multivariate Cox regression analysis, the high-risk group had a higher risk of death (HR, 3.89; 95% CI, 1.02–14.84; *P =* 0.047; **Figure 4B** and **Supplementary Tables 3, 4**). The nomogram had high performance in the validation group to predict the risk of death during induction therapy (AUC was 0.85 for the death in 4 weeks and was 0.81 for the death in 8 weeks**; Figures 4C, D** and **Table 3**). The calibration curves showed that the death predicted by the nomogram was consistent with the actual death (**Figures 4E, F**).

We further performed the subgroup analysis based on the therapy strategy (**Table 4**). The nomogram was used to categorize all patients of the primary and validation groups into the low-risk group (n = 177) and the high-risk group (n = 57). For those patients treated with etoposide, the mortality was 43.9 per 100 person-weeks for the high-risk patients, significantly higher than the mortality of 3.1 per 100 person-weeks for the low-risk patients. For the therapy without etoposide, the result was similar (34.4 vs. 9.5, per 100 person-weeks). The mortality was higher in the second 4-week period.

**Table 4:** The incident rate of death in patients receiving different therapy strategy.

Groups		Etoposide-basedn = 181		No etoposide-basedn = 53	
		no.^†^	IR (95% CI)^††^	no.^†^	IR (95% CI)^††^
Low-risk group^†††^ n = 177	The first 4 weeks	4	2.9 (0.1–5.7)	4	10.8 (0.3–21.3)
	The second 4 weeks	6	4.4 (0.9–7.9)	3	9.1 (0.0–19.4)
	Overall	10	3.1 (1.4–5.8)	7	9.5 (2.9–16.1)
High-risk group^†††^ n = 57	The first 4 weeks	18	43.9 (28.0–59.8)	7	43.8 (16.4–71.1)
	The second 4 weeks	18	78.3 (60.0–96.5)	4	44.4 (3.9–85.0)
	Overall	36	43.9 (38.7–49.2)	11	34.4 (21.6–47.2)

^†^The accumulative number of deaths.

^††^The incident rate of death per 100 person-weeks.

^†††^ The patients with nomogram points > 80 were recognized as low-risk group, otherwise as the high-risk group.

IR, incident rate.

## Discussion

EBV-HLH is a second type of HLH with high mortality and has a high incidence in Asians (1, 8). Significant mortality in induction therapy has been found as a significant cause for poor outcome (1–3). In this study, we found age, Bun, PCT, sCD25, and EBV-DNA in PBMC as the factors of death during induction therapy. On the basis of the above factors, we built a nomogram model and validated the prediction accuracy of this model for the death in induction therapy. In the subgroup analysis, the high-risk patients had a higher mortality in both patients treated with etoposide and patients not treated with etoposide. High-risk patients treated with etoposide had a lower mortality.

This study found that patients with the age >18 years, increased sCD25, and high EBV-DNA in PBMC had a higher risk of death in induction therapy. For EBV-related diseases, older age was considered a sign of poor outcome (4). As reported in a previous study, the child patients were more likely to have a good outcome (9, 10), and the age ≥50 was a predictor for poor outcome (6), probably because mature immune system of adult patients can cause a severer inflammation response compared with the immature immune system of child patients (11, 12). sCD25 is positively correlated with the systematic inflammation response, which was recognized as a parameter to monitor the recurrence of HLH (13). Compared with the secondary HLH caused by other factors, patients with EBV-HLH had a higher sCD25 level (14). The 5-year survival possibility of patients with HLH with sCD25 >10,000 IU/ml was 36%, significantly lower than that of patients with sCD25 ≤10,000 IU/ml as 78% (5). Thus, the high sCD25 level indicated that patients with EBV-HLH had a severe condition. Moreover, high EBV-DNA in PBMC was found as the factor of death in induction therapy, which was consistent with existing studies (10, 15). We considered that it might be because high EBV-DNA can induce a severe T cell and NK cell response. The EBV-DNA in PBMC reflects the intracellular EBV load, whereas the EBV-DNA in plasma reflects the leakage virus, which is from cells and tissues necrotic (16). For different EBV-related diseases, the EBV-DNA in plasma and in PBMC had different diagnostic values. For example, like chronic active EBV infection, EBV-DNA in PBMC had a higher predictive value for prognosis (17). However, for EBV-related tumor, like EBV+ NK/T cell lymphoma, EBV-DNA in plasma is meaningful (18). The pathological mechanism of EBV-HLH is close to chronic active EBV infection. For EBV-HLH, EBV-infected cells cannot be eliminated, which will result in an abnormal increase of cytokines (19). More EBV existed in NK/T cells, with no leak into plasma. Thus, for EBV-HLH, EBV-DNA in PBMC had a higher predictive value for prognosis.

Importantly, we found that high Bun and PCT >2 µg/L were correlated with a high risk of death in induction therapy. The increased Bun reflected impaired renal function and multiple organs dysfunctions. Existing studies reported a high incident rate of acute kidney injury in the activity period of HLH, which was mainly caused by acute renal tubular necrosis, renal hypoperfusion, tumor lysis syndrome, and HLH-related glomerulopathy (20). In addition to HLH, chemical therapy can cause renal injury and a multiple-organ failure. In this study, we found that the risk of death would increase 1.08 times if the Bun increased as 1 U/L. Thus, a high Bun level before induction therapy can indicate the risk of death. Interestingly, PCT >2 µg/L was correlated with the high risk of death as well. In patients with the secondary HLH, an increased PCT was found in 77.7% of those patients (21). PCT >2 µg/L is correlated with a severe infection, especially bacterial infection, which is an important cause of death of patients with HLH (22, 23). During induction therapy, the immunological suppression caused by chemotherapy will increase the risk of bacterial infection (24). As indicated by our result, the patients with PCT >2 µg/L had 2.6 times of death compared with the patients with PCT ≤2 µg/L. Of the 19 patients who died with PCT >2 µg/L, 10 patients (52.6%) died of severe infection. Accordingly, PCT is also a parameter to predict the risk of death.

By integrating age, Bun, sCD25, and EBV-DNA in PBMC, we developed a nomogram to more effectively find the high-risk patients. This nomogram transferred patients’ characteristics into points and categorized patients into the high-risk group and the low-risk group based on the above points, which is easily accessible to physicians. As indicated by our result, this nomogram had higher prediction accuracy compared with single parameters. If a patient has the nomogram points >80, then this patient will be recognized as belonging to the high-risk group, with a death probability in 4 weeks of 30% and a death probability in 8 weeks of more than 50%. We should monitor the apostasies (e.g., multiple organ dysfunction syndrome, severe infection, or gastrointestinal bleeding) once chemical therapy was admitted, especially in the second 4 weeks after patients received therapy. For patients with the nomogram points <80, they were assigned to the low-risk group and had a favorable outcome during induction therapy.

Another notable finding is the different therapeutic responses to different therapy strategies. A good outcome was found in low-risk patients not treated with etoposide and high-risk patients treated with etoposide during induction therapy. The reason for this outcome was considered to be that high-risk patients usually had a severer condition of overactivated immune cells compared with low-risk patients. Etoposide can quickly kill the overactivated immune cells, which is conducive to controlling EBV-HLH (4). However, low-risk patients could be quickly relieved after simple immunosuppressive therapy, e.g., corticosteroids (7). Thus, the therapy with etoposide is urgently recommended for high-risk patients.

This study had some limitations. First, this study was a single-center and retrospective study. Our conclusion might be limited by the cross-sectional study design. Second, we only investigated the risk factors of death in induction therapy and the reliability of the nomogram based on Chinese population. Whether our conclusion is suitable for other populations is unclear. Third, we only included the results of laboratory examination before induction therapy and did not monitor the change of each laboratory parameter during induction therapy. Fourth, we did not discuss the effect of EBV infection cell type on outcome. Although this study had the above limitations, we still developed a useful nomogram model to identify the high-risk patients with EBV-HLH before induction therapy.

## Conclusion

Age, Bun, PCT, sCD25, and EBV-DNA in PBMC were the factors for death during induction therapy. We developed a nomogram to help identify the patients with a high risk of death during induction therapy. For patients with different risks of death, the therapy strategy could be different to improve the outcome during induction therapy. The therapy with etoposide is recommended for high-risk patients. It is necessary to monitor the apostasies of the mentioned high-risk patients, especially in the second 4 weeks after they receive chemical therapy. Acknowledgments

We thank Dr. Qingyuan Liu (China National Clinical Research Center for Neurological Diseases and Beijing Tiantan Hospital, Beijing, China) for helping in statistical analyses.

## Data Availability Statement

The original contributions presented in the study are included in the article/**Supplementary Material**. Further inquiries can be directed to the corresponding author/s.

## Ethics Statement

The studies involving human participants were reviewed and approved by the Institutional Review Board of Beijing Friendship Hospital. Written informed consent to participate in this study was provided by the participants’ legal guardian/next of kin.

## Author Contributions

Author contributions to the study and manuscript preparation include the following. Conception and design: ZW and JW. Acquisition of data: TC. Analysis and interpretation of data: TC. Drafting the article: TC. Critically revising the article: ZW. Approving the final version of the manuscript on behalf of all authors: ZW. Study supervision: ZW. All authors contributed to the article and approved the submitted version.

## Funding

This study was supported by the “National Natural Science Foundation of China (Grant No. 81871633 and 82170122)” and “The Capital Health Research and Development of Special (Grant No. 2020-1-2022)”.

## Conflict of Interest

The authors declare that the research was conducted in the absence of any commercial or financial relationships that could be construed as a potential conflict of interest.

## Publisher’s Note

All claims expressed in this article are solely those of the authors and do not necessarily represent those of their affiliated organizations, or those of the publisher, the editors and the reviewers. Any product that may be evaluated in this article, or claim that may be made by its manufacturer, is not guaranteed or endorsed by the publisher.

## Abbreviation

HLH, hemophagocytic lymphohistiocytosis; EBV, Epstein–Barr virus; sCD25, serum CD25; PBMC, peripheral blood mononuclear cell; ALT, glutamic-pyruvic transaminase; AST, glutamic oxalacetic transaminase; GGT, gamma-glutamyl transpeptidase; ALP, alkaline phosphatase; Tbi, total bilirubin; Dbi, direct bilirubin; Ibi, indirect bilirubin; ALB, albumin; Cr, creatinine; Bun, blood urea nitrogen; PCT, procalcitonin; ESR, erythrocyte sedimentation rate; NK cells, nature killer cells.
