# Supplementary material for: The Outcome of Induction Therapy for EBV-Related Hemophagocytic Lymphohistiocytosis: A Model for Risk Stratification
Source: Front Immunol. 2022 Jul 4;13:876415. doi: 10.3389/fimmu.2022.876415 (PMC9289144; doi:10.3389/fimmu.2022.876415)
Supplement: Supplementary file 1 [file DataSheet_1.pdf]

Supplementary materials

Supplementary materials to *The outcome of induction treatment in Epstein-Barr virus related hemophagocytic lymphohistiocytosis patients: a nomogram model to risk assessment*

Supplementary table 1. Univariate Cox regression analysis for the risk factors associated with the death in induction treatment based on primary cohort

	HR	95% CI	*P* value
Male	1.53	0.89-2.61	0.121
Age >18 years	1.48	1.04-2.13	0.031┼
Etoposide-based strategy	1.52	0.83-2.79	0.181
Tuberculous infection history	1.89	1.00-3.60	0.051
Brain involvement	1.14	0.63-2.08	0.659
Hepatosplenomegaly	0.93	0.65-1.34	0.705
WBC count	0.97	0.91-1.03	0.280
Hemoglobin	1.00	0.99-1.01	0.595
Platelet count	1.00	1.00-1.00	0.483
GGT	1.00	1.00-1.00	0.040┼
ALP	1.00	1.00-1.00	0.067
Tbi	1.00	1.00-1.00	0.160
Dbi	1.00	1.00-1.00	0.220
Cr	1.01	1.00-1.01	0.003┼
Bun	1.08	1.03-1.13	0.003┼
ALB	0.98	0.95-1.00	0.085
Ca^{2+}	0.67	0.32-1.37	0.268
Na^{+}	0.97	0.94-1.01	0.144
Fibrinogen	1.00	1.00-1.00	0.102
PCT >2 ug/L	1.05	1.01-1.10	0.028┼
sCD25	1.07	1.02-1.12	0.004┼
EBV-DNA in PBMC	1.09	1.04-1.14	<0.001┼

┼, the parameter was significant.

Abbreviation: HR, hazard ratio; WBC, white blood cell; GGT, gamma-glutamyl transpeptidase; ALP, alkaline phosphatase; Tbi, total bilirubin; Dbi, direct bilirubin; ALB, albumin; Cr, creatinine; Bun, blood urea nitrogen; PCT, procalcitonin; serum CD25; EBV, Epstein-Barr virus; DNA, deoxyribonucleic acid; PBMC, peripheral blood mononuclear cell; CTL, cytotoxic T lymphocyte.

Supplementary table 2. Demographic and clinical information of EBV-HLH patients in the validation cohort

Characteristics	Death n=10	Survival n=40	*P* value
Group categorized by nomogram – no. (%)			0.030†
High-risk group	5 (50.0%)	6 (15.0%)	
Low-risk group	5 (50.0%)	34 (85.0%)	
Male – no. (%)	7 (70.0%)	23 (57.5%)	0.475
Age >18 years – no. (%)	10 (100.0%)	28 (70.0%)	0.049†
Treatment strategy – no. (%)			0.548
Etoposide-based	6 (60.0%)	28 (70.0%)	
No etoposide-based	4 (40.0%)	12 (30.0%)	
Infection history – no. (%)			
Tuberculous	2 (20.0%)	1 (2.5%)	0.039†
Other	8 (80.0%)	39 (97.5%)	1.000
Brain involvement – no. (%)	1 (10.0%)	3 (7.5%)	0.796
Liver/spleen involvement – no. (%)			0.182
Splenomegaly	0 (0.0%)	1 (2.5%)	
Hepatomegaly	6 (60.0%)	13 (32.5%)	
Hepatosplenomegaly	4 (40.0%)	26 (65.0%)	
Laboratory findings			
WBC count – m (IQR) – $\times 10^9$	2.1 (1.3-3.0)	1.9 (1.4-3.1)	0.884
Hemoglobin (g/l) – m (IQR)	108 (101-122)	97 (79-114)	0.075
platelet count – m (IQR) – $\times 10^9$	81.5 (28.0-164.0)	72.5 (31.5-125.5)	0.762
ALT (U/l) – m (IQR)	138.6 (51.2-370.0)	93.5 (53.0-255.0)	0.846
AST (U/l) – m (IQR)	261.7 (68.5-286.5)	87.3 (46.8-195.0)	0.174
GGT (U/l) – m (IQR)	132 (79-342)	164.5 (58.5-281)	0.725
ALP (U/l) – m (IQR)	406.5 (118.0-619.0)	203.0 (129.0-573.0)	0.482

Tbi (μmol/l) – m (IQR)	26.0 (9.8-202.1)	18.7 (11.6-40.1)	0.309
Dbi (μmol/ l) – m (IQR)	15.2 (1.9-100.0)	3.8 (2.1-15.0)	0.152
Ibi (μmol/ l) – m (IQR)	11.3 (7.9-34.1)	11.3 (7.6-19.1)	0.314
ALB (g/l) – m (IQR)	29.4 (25.7-33.7)	30.0 (26.1-36.8)	0.389
Cr (μmol/l) – m (IQR)	50.2 (37.9-77.5)	47.8 (32.9-58.3)	0.452
Bun (mmol/l) – m (IQR)	4.3 (3.5-8.3)	4.9 (3.6-7.7)	0.619
K^+ (mmol/l)– m (IQR)	4.0 (3.8-4.1)	4.0 (3.8-4.2)	0.846
Ca^{2+} (mmol/l) – m (IQR)	2.0 (1.9-2.1)	2.1 (2.0-2.2)	0.409
Na^+ (mmol/l) – m (IQR)	134.4 (128.1-137.9)	137.6 (135.4-138.9)	0.049†
Fibrinogen (g/l) – m (IQR)	1.42 (1.20-2.32)	1.42 (1.06-1.88)	0.671
PCT >2 ug/l – no. (%)	6 (60.0%)	37 (92.5%)	0.009†
ESR (mm/H) – m (IQR)	19 (8-47)	14 (9.5-24.5)	0.510
Specific findings			
Hemophagocytosis – no. (%)	6 (60.0%)	29 (72.5%)	
Serum ferritin (ng/ml) – m (IQR) – $\times10^3$	2.4 (1.3-11.6)	1.6 (1.0-12.2)	0.420
sCD25 (pg/ml) – m (IQR) – $\times10^4$	3.7 (1.0-4.4)	3.4 (1.4-4.4)	0.639
The activity of NK cells (%) – m (IQR)	13.9 (10.1-17.4)	14.6 (12.8-16.9)	0.670
EBV-DNA (copies/ml) – m (IQR), $\times10^5$			
Plasma	1.0 (0.1-3.1)	0.1 (0.1-9.7)	0.464
PBMC	8.7 (1.1-31.0)	5.8 (1.6-29.0)	0.884
NK-CD107a (%) – m (IQR)	10.5 (7.2-27.8)	15.4 (8.6-24.3)	0.458
CTL-CD107a (%) – m (IQR)	5.4 (2.8-8.1)	3.3 (2.2-4.5)	0.231

†, the parameter was significant.

Abbreviation: WBC, white blood cell; ALT, glutamic-pyruvic transaminase; AST, glutamic oxalacetic transaminase; GGT, gamma-glutamyl transpeptidase; ALP, alkaline phosphatase; Tbi, total bilirubin; Dbi, direct bilirubin; Ibi, indirect bilirubin; ALB, albumin; Cr, creatinine; Bun, blood urea nitrogen; PCT, procalcitonin; ESR, erythrocyte sedimentation rate; serum CD25; NK cells, nature killer cells; EBV, Epstein-Barr virus; DNA, deoxyribonucleic acid; PBMC, peripheral blood mononuclear cell; CTL, cytotoxic T lymphocyte.

Supplementary table 3. Univariate Cox regression analysis for the risk factors associated with the death in induction treatment based on the primary cohort

	HR	95% CI	*P* value
Group categorized by nomogram			0.012┼
High-risk group	4.93	1.42-17.11	
Low-risk group	0.20	0.06-0.71	
Age >18 years	-	-	-
Tuberculous infection	3.96	0.84-18.77	0.083
Etoposide-based strategy	1.53	0.43-5.44	0.509
Bun	0.99	0.82-1.19	0.893
PCT >2 ug/L	6.96	1.94-25.03	0.003┼
sCD25	1.00	1.00-1.00	0.833
EBV-DNA in PBMC	1.00	1.00-1.00	0.138

-, the parameter couldn't be calculated.

┼, the parameter was significant.

Abbreviation: HR, hazard ratio; PCT, procalcitonin; serum CD25; EBV, Epstein-Barr virus; DNA, deoxyribonucleic acid; PBMC, peripheral blood mononuclear cell.

Supplementary table 4. Multivariate Cox regression analysis for the risk factors associated with the death in induction treatment based on validation cohort

	HR	95% CI	*P* value
Group categorized by nomogram			0.047
High-risk group	3.89	1.02-14.84	
Low-risk group	0.26	0.07-0.98	
Treatment strategy			0.629
Etoposide-based	1.43	0.33-6.19	
No etoposide-based	0.70	0.16-3.01	
Tuberculous infection	3.20	0.53-19.29	0.204

Abbreviation: HR, hazard ratio.
